# Endoscopic Push-Through Cartilage Myringoplasty for Anterior Perforations

**DOI:** 10.22038/IJORL.2023.63643.3181

**Published:** 2023-03

**Authors:** Ankita Aggarwal, Sanjeev Bhagat, Dimple Sahni, Dinesh Kumar Sharma, Vishav Yadav

**Affiliations:** 1 *Department of Otolaryngology, Head and Neck Surgery, Government Medical College Patiala, India.*

**Keywords:** Cartilage, Endoscopy, Myringoplasty, Tympanic membrane perforation

## Abstract

**Introduction:**

The study aims to evaluate the anatomical and functional success rates of endoscopic push-through cartilage myringoplasty for anterior tympanic membrane (TM) perforations.

**Materials and Methods:**

Thirty patients with TM perforations in the anterior quadrant underwent endoscopic push-through cartilage tympanoplasty and underwent prospective evaluation. The graft uptake rate and hearing gain were the outcomes evaluated.

**Results:**

Out of the 30 patients, 15 were male, and 15 were female. The mean age was 32.60 ± 13.66 years (from 18-60 years). The overall graft uptake rate was 90%, with failure observed in three cases. The mean preoperative air conduction threshold was 37.9 ± 5.83 dB which improved to 27.66 ± 4.88 dB at 16 weeks post-operation. The mean postoperative ABG closure was 7.28 dB with a p-value of 0.001 which was statistically significant.

**Conclusions:**

Endoscopic push-through cartilage myringoplasty is the least invasive, safe, simple, and advantageous for healing TM perforation and hearing restoration.

## Introduction

Chronic otitis media (COM) is an inflammation of the middle ear cavity that often leads to permanent changes in the tympanic membrane (TM), namely atelectasis, perforation, tympano- sclerosis, retraction pocket development, and formation of cholesteatoma (1). 

Myringoplasty is the standard procedure performed to close the TM perforation to avoid frequent otorrhea and to restore the sound-conducting mechanism (2). Repairing anterior quadrant TM perforations remains a challenge for the surgeon. This could be explained due to the anterior canal wall bulge, because of which anterior perforation margins and annulus are not visualized completely. In addition, anterior stabilization is inappropriate due to the buildup of negative pressure by sniffing and the reduced vascular supply, which further declines the probability of graft viability (3). 

Among the various graft materials used, cartilage grafts are preferred due to their durability, stability, low metabolism, and resistance (4). Also, they have been used widely in revision surgery cases with promising results (5). Recently, endoscopy has gained attention among ENT surgeons. In the novel endoscopic “push-through” technique, the cartilage graft is pushed through the freshened perforation by applying a trans-canal underlay approach without any tympanomeatal flap elevation. Thus, the trans-canal endoscopic approach does not require surgical exposure *via* retro auricular skin incision, hence avoiding tissue dissection. In addition, except for graft harvesting, no external incision is required, allowing for better cosmesis (6). Further, endoscopes avoid the ear canal overhangs, eliminating the need for anterior canaloplasty and shortening the surgical procedure (7). Research on endoscopic cartilage push-through myringoplasty with anterior eardrum perforations is scarce. Hence, our study aimed to evaluate the results of this technique in 30 COM patients with perforations of the anterior TM. The outcomes evaluated were based on graft success rate and hearing gains.

## Materials and Methods

A prospective study was conducted on 30 patients with clinically diagnosed cases of COM with anterior quadrant TM perforation.

*Inclusion criteria:* Patients of both sexes of 18 to 60 years with anterior quadrant (anterosuperior, anteroinferior) TM perforation, who had a dry ear for the previous three months and air-bone gap of ≤25 dB in the pre-operative audiogram were included. Revision cases with residual perforation in the anterior quadrant were also included. 

*Exclusion criteria:* Patients with posterior quadrant TM perforations, an air-bone gap of >25 dB in pre-operative audiogram, and a cholesteatoma.*Protocol:* The study protocol was approved by the local institutional ethics committee (8(109)2019/7856), and written informed consent was obtained from all patients. Patients fulfilling the inclusion criteria were admitted one day before surgery. Past medical history was noted, and a complete ENT examination was done. Pure tone audiometry (PTA) and lateral mastoid radiographs/HRCT temporal bone were performed.

Surgery was conducted under local anesthesia (LA), and premedication was administered with 0.4 mg atropine and 25 mg promethazine intramuscularly (IM) two hours before surgery. Topical anesthesia (2% lidocaine, 1:100,000 epinephrine) was injected into the four quadrants of the external ear canal. A tragal perichondrial composite cartilage graft (with perichondrium freed from one side) was collected intraoperatively from all patients.

**Fig 1:**
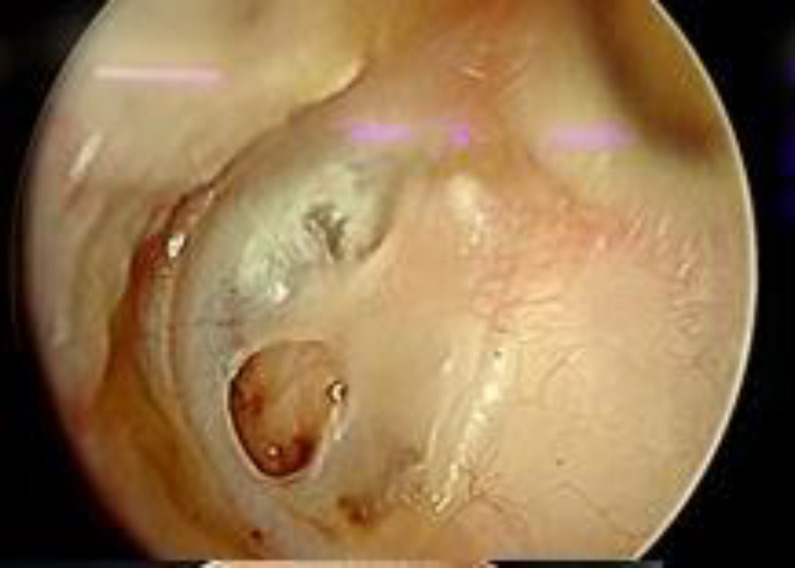
Preoperative otoendoscopic picture showing anterior tympanic membrane perforation

The harvested tragal cartilage was used in its normal thickness, and no cartilage slicing was done. Non-absorbable suture material was used to close the tragal site. A cartilage graft was prepared, which was 2-3mm larger than the perforation. The perforation edges and anterior annulus were visualized *via* the trans-canal route using a 0° Hopkins rigid endoscope (Fig 1). 

**Fig 2:**
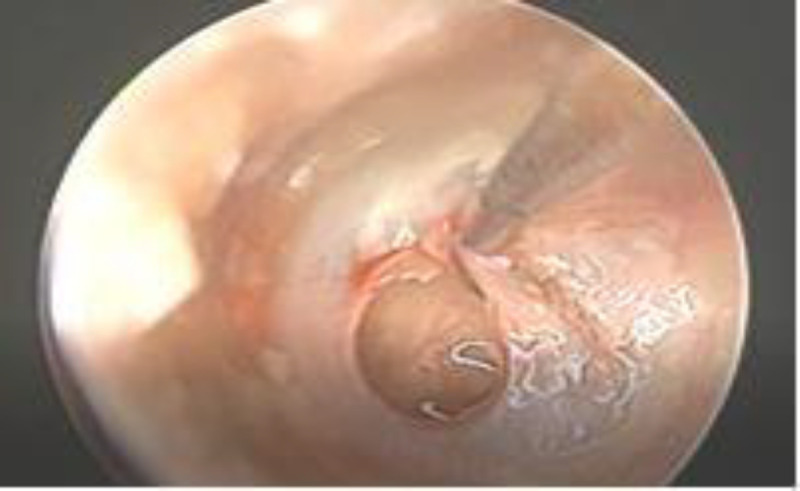
Freshening of the anterior perforation margins

**Fig 3:**
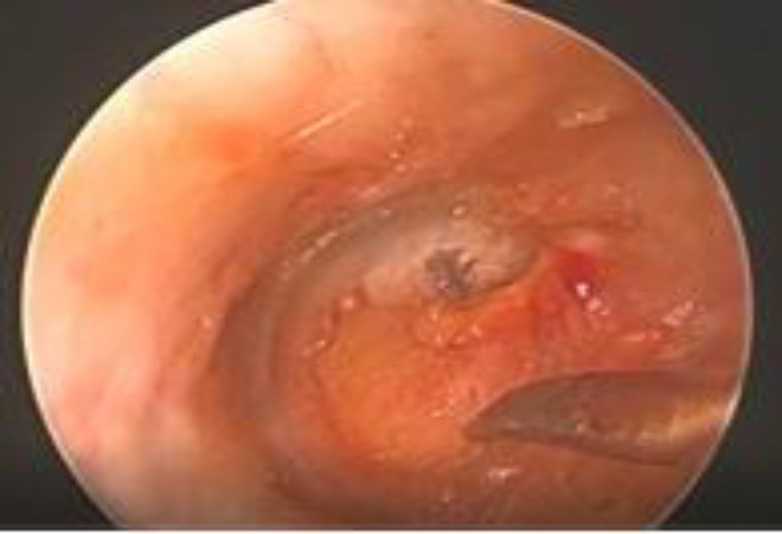
Cartilage graft being pushed through the tympanic membrane perforation

The perforation edges (Fig. 2) and the mucosal undersurface were made raw. The middle ear was packed with gel foam, and the graft was pushed through the perforation (Fig. 3) and placed in an underlay fashion with its concave and perichondrium-free surface facing the middle ear cavity. It was then covered with gel foam till it reached the level of isthmus. 

**Fig 4:**
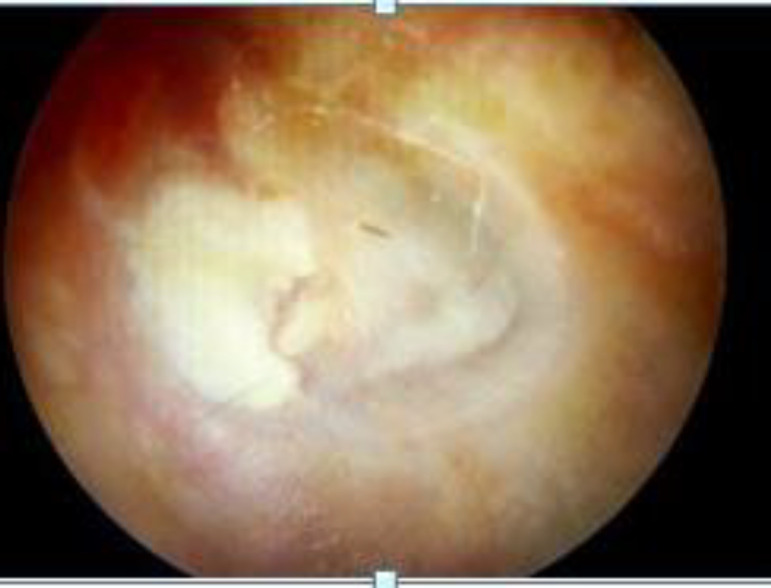
Postoperative otoendoscopic picture at 16 weeks showing graft uptake

Patients were discharged on the same day. All patients received antibiotics, antihistamines, and analgesics as needed post-operation. The patients were instructed to keep the operated ear dry and avoid straining. Skin sutures (tragal) were removed seven days post-surgery, and antibiotic ear drops were given.The packing material (gel foam) was entirely suctioned out of the EAC 4 weeks after the surgical procedure. All cases were followed up for at least four months. Follow-up of the patients was conducted at six weeks, 10 weeks, and 16 weeks to evaluate the status of graft uptake using otoendoscopy (Fig. 4). 

Pre-operative and post-operative PTA measurements were calculated and scored at four frequencies (0.5, 1, 2, and 4 kHz) based on the recommendations of the American Academy of Otolaryngology-Head and Neck Surgery Committee (AAO-HNS).

## Results

  The study enrolled 30 patients with small to medium anterior TM perforations (15 men and 15 women). The age of the patients varied from 18 to 60 years, with a mean age of 32.60 ± 13.66 years. Maximum cases (56.67%) presented with anteroinferior (AI) quadrant perforation, with 10% of cases having anterosuperior (AS) quadrant perforation and 33.3% with medium-sized (AS+AI) anterior perforations. 

 The most common complaint was ear discharge with hearing loss in 53.3%, followed by ear discharge alone in 36.6% of patients and hearing loss alone in 10% of patients. 

 Out of 30 patients, three underwent revision surgery. By the end of four months, the graft uptake rate was 90%, with graft failure occurring in three patients. 

 Amongst these, two patients had graft medialization, one at the inferior edge and another at the posterior edge, respectively, and one patient reported reperforation, post operation. Minor complications such as secondary otomycosis were reported in two of the post-operative patients and one patient who had reperforation also suffered from otitis externa, all of which were treated conservatively. PTA was calculated using AC thresholds at 0.5, 1, 2, and 4 kHz (according to AAO-HNS criteria). Student’s paired t-test was applied to collect the data and analyze the acoustic gain at various pre-specified intervals (p-value <0.05~significant).

 Preoperative mean PTA was 37.97 ± 5.83 dB, which improved to 27.66 ± 4.88 dB at 16 weeks postoperatively. The mean difference was 10.31 ± 3.82 with a significant p-value (0.001). The mean preoperative ABG (AB gap) was 18.58 ± 2.69 dB, and the mean post-operative ABG was 11.30 ± 4.09dB with post-operative ABG closure of 7.28 ± 3.37 dB, which was statistically significant.

## Discussion

  The repair of TM perforations has seen the widespread use of several novel procedures. In contrast to the conventional approach, our endoscopic “push through” technique is unique as cartilage is pushed through the fresh perforation edges without the tympanomeatal flap elevation. In addition, endoscopes provide a magnified view and better illumination and are easy to handle while avoiding the need for canaloplasty (6).

Cartilage grafts are favored over other materials for repairing anterior TM perforations. The highlighting features are its low metabolism, resistance, and good receptivity in the middle ear (8). Further, tragal cartilage perichondrial graft is simple to yield because it is located within the surgical field and does not require hair removal or wide dissection (9). In our study, tragal cartilage was applied at its full thickness, and no thinning was done. A comparative study by Elfouly, M.S. showed that at postoperative 1 year of tympanoplasty, the difference in ABG gain between the group1 (full thickness tragal cartilage) versus the group2 (sliced mosaic cartilage) was statistically insignificant (10). Further, Vadiya S compared tympanoplasty outcomes in group A (partial thickness tragal cartilage) and group B (full thickness tragal cartilage). PTA showed no significant difference in acoustic gain between these two groups (except at 4,000 Hz) four months post-surgery (11). Based on past literature, the graft uptake rate varies between 84.4 - 97.4% in patients undergoing endoscopic push-through myringoplasty rate (3,12-14). 

In our study of endoscopic cartilage push-through technique for anterior perforations, the graft uptake success rate was evaluated and found to be 90% (27 out of 30) at 16 weeks post operation. Graft failure was observed in three patients, and two patients had graft medialization at the inferior and posterior edge, respectively, while one patient reported re-perforation. Similarly, Celik *et al.* reported successful graft uptake of 87.5% (28 of 30) with this technique at four months post operation. Revision surgery was done by fat myringoplasty in one patient and another patient had spontaneous closure in the 6th post-operative month. Therefore, only two patients were categorized as graft failures, and no minor or major complications were reported except for one patient with a cholesteatoma pearl (3).

El Hennnawi *et al.* compared the endoscopic push-through technique with microscopic underlay myringoplasty. The microscopic underlay myringoplasty group had a graft uptake rate of 85.7% (24/28 patients), compared to 92.9% (26/ 28 patients) in the endoscopic trans-canal push-through myringoplasty group. They documented similar findings to our study, i.e. small pin-point perforation at the anterosuperior margin of the graft in two patients post-operatively (12). S Gulsen *et al.* compared endoscopic butterfly inlay and endoscopic push-through myringoplasty for repairing anterior perforations. The success rates of endoscopic butterfly-inlay cartilage myringoplasty and endoscopic push-through myringoplasty were 94.1% (32/34 patients) and 91.8% (34/37 patients), respectively (p > 0.05). In the endoscopic push-through myringoplasty group, medialization in two patients and reperforation in one patient were the causes of graft failure (4). Zhengcai Lou *et al.* reported that the graft uptake rate was 95.74% (45/47) in the push-through cartilage group at the post-operative 12th month, and the re-perforation rate was 4.26% (9). Thus, the failure rate of the endoscopic cartilage push-through technique varied between 4-15% (3,4,9,14,15). In most cases, re-perforation occurred at the graft edge, most likely due to inadequate cartilage grafts. A dysfunctional Eustachian tube could also be responsible for this re-perforation. (9). 

According to Mouna *et al.,* there was no statistically significant difference in audiological outcomes between fascia and cartilage grafts (16). In our study, we observed the preoperative mean PTA to be 37.97 ± 5.83dB which improved to 27.66 ± 4.88 dB at 16 weeks post-operation. The post-operative ABG closure (AB gap) was 7.28±3.37 dB which was statistically significant (p-value- 0.001). According to Celik *et al.,* the preoperative mean air conduction threshold improved to 19.5 dB and the mean ABG improved from 11.9 dB to 5.3 dB postoperatively with a significant p-value of 0.001 (3). Correspondingly in a study by El Hennawi *et al*, the mean (± SD) preoperative air-bone gap was 17.4 ± 3.7 dB in the endoscopic trans-canal push-through myringoplasty group and 18.5 ± 2.2 dB in the microscopic underlay myringoplasty group. The air-bone gap was significantly improved in both groups at the end of the follow-up period, with values in the endoscopic trans-canal push-through myringoplasty group at three months being 6.1 ± 3.7 dB (12).

In another study by S Gulsen *et al.,* the functional results of endoscopic butterfly-inlay cartilage myringoplasty and endoscopic push-through myringoplasty were evaluated. The pre and post-operative mean (± SD) air-bone gap values of the patients undergoing endoscopic push-through myringoplasty were 18.2 ± 5.1 dB and 6.1 ± 3.6 dB, respectively ( p < 0.001), which was again similar to our study (4). Thus, these results from various studies were consistent with our study (Table 1).

**Table 1:** Results of various studies on endoscopic cartilage push-through myringoplasty in anterior perforations

**Sr. No.**	**Author**	**No. of patients**	**Year of Study**	**Graft uptake**	**Pre-op ABG (dB)**	**Post-op ABG (dB)**	**P value** **<0.05=significant**
1.	Hatice Celik(3)	32	2015	87.5%	11.9±4.8	5.3±3.8	P=0.001
2.	El Hennawi (12)	28	2018	92.9%	17.4	6.1	P<0.05
3.	Gulsen S,Erden B(4)	37	2020	91.8%	18.2±5.1	6.1±3.6	P<0.001
4.	Present study	30	2020	90%	18.58±2.69	11.3±4.09	P=0.001

Hence, the advantages of endoscopic cartilage push-through myringoplasty over other techniques include less bleeding, using local anesthesia for surgery to reduce the risks of general anesthesia, better cosmesis, daycare surgery, patient comfort, and ease of performing in residual perforations.

However, a few disadvantages are its single-handedness, stereoscopic view, and recurrent fogging, which makes it technically difficult (17).

## Conclusion

  Endoscopic push-through cartilage myringoplasty for anterior perforation is safe and effective for anatomic healing and audiological outcomes. Hence, more studies are encouraged to apply this approach to establish a stronger basis.

 However, the limitations of our study include a small sample size (30 patients) and a short follow-up period (four months) without a control group. A larger sample size with a longer duration of follow-up is required to evaluate the long-term results of the push-through cartilage technique.

## References

[1] Glasscock ME, ShambaughGE (2010). Surgery of the Ear.

[2] Jain S, Gupta N, Gupta R, Roy A (2017). Interlay Type I tympanoplasty in large central perforations: Analysis of 500 cases. Indian J Otol..

[3] Celik H, Samim E, Oztuna D (2015). Endoscopic" push-trough" technique cartilage myringoplasty in anterior tympanic membrane perforations. Clinical and experimental otorhinolaryngology..

[4] Gülşen S, Erden B (2020). Comparison of endoscopic butterfly-inlay versus endoscopic push-through myringoplasty in repairing anterior perforations of the tympanic membrane. The Journal of Laryngology & Otology..

[5] Duckert LG, Mueller J, Makielski KH, Helms J (1995). Composite autograft shield reconstruction of remnant tympanic membranes. Am J Otol..

[6] Ballal VC, Shivappa L, Tmmappaiah SB, Basavannaiah S, Rangaswamy CT (2019). Minimally invasive cartilage myringoplasty: our technique and experience. International Journal of Otorhinolaryngology and Head and Neck Surgery..

[7] Jako GJ (1967). Postaural versus endaural exposure in tympanoplasty. Laryngoscope.

[8] Yung M (2008). Cartilage tympanoplasty: Literature review. J Laryngol Otol..

[9] Lou Z (2020). Endoscopic myringoplasty in pediatric patients: a comparison of cartilage graft push-through and underlay fascia graft techniques. Acta Otolaryngol.

[10] Elfouly M S (2021). Full thickness vs sliced mosaic cartilage graft in tympanoplasty: a comparative study. Egypt J Otolaryngol.

[11] Vadiya S, Bhatt S (2016). Comparison of Partial Thickness and Full Thickness Tragal Cartilage Graft During Modified Cartilage Shield Tympanoplasty for Type I Procedures. Indian J Otolaryngol Head Neck Surg..

[12] El-Hennawi DEM, Ahmed MR, Abou-Halawa AS, Al-Hamtary MA (2018). Endoscopic push-through technique compared to microscopic underlay myringoplasty in anterior tympanic membrane perforations. J Laryngol Otol.

[13] Tseng CC, Lai MT, Wu CC, Yuan SP, Ding YF (2018). Endoscopic transcanal myringoplasty for tympanic perforations: an outpatient minimally invasive procedure. Auris Nasus Larynx.

[14] Loh TL, Ranguis S, Patel H, Crossland G (2018). Permeatal ‘push through’ myringoplasty in the Northern Territory: a prospective cohort. Aust J Otolaryngol.

[15] Simsek E, Ozkan O, Kucur C, Carlıoglu A (2016). Evaluation of the anatomical and auditory outcomes of minimally invasive cartilage myringoplasty: Our technique and experience. American journal of otolaryngology..

[16] Mouna B, Khalifa M, Ghammem M, Limam M, Meherzi A, Kermani W (2019). Cartilage and fascia graft in type 1 tympanoplasty: comparison of anatomical and audiological results. J Craniofac Surg.

[17] Tarabichi M, Nogueira JF, Marchioni D, Presutti L, Pothier DD, Ayache S (2013). Transcanal endoscopic management of cholesteatoma. Otolaryngol Clin North Am.

